# A Novel Isolate of Spherical Multicellular Magnetotactic Prokaryotes Has Two Magnetosome Gene Clusters and Synthesizes Both Magnetite and Greigite Crystals

**DOI:** 10.3390/microorganisms10050925

**Published:** 2022-04-28

**Authors:** Kaixuan Cui, Hongmiao Pan, Jianwei Chen, Jia Liu, Yicong Zhao, Si Chen, Wenyan Zhang, Tian Xiao, Long-Fei Wu

**Affiliations:** 1 CAS Key Laboratory of Marine Ecology and Environmental Sciences, Institute of Oceanology, Chinese Academy of Sciences, Qingdao 266071, China; 18661379736@163.com (K.C.); panhongmiao@qdio.ac.cn (H.P.); liujia@qdio.ac.cn (J.L.); zyc15627861591@163.com (Y.Z.); chensi0096@foxmail.com (S.C.); 2 Laboratory for Marine Ecology and Environmental Science, Pilot National Laboratory for Marine Science and Technology (Qingdao), Qingdao 266237, China; 3 University of Chinese Academy of Sciences, Beijing 100049, China; 4 Center for Ocean Mega-Science, Chinese Academy of Sciences, Qingdao 266071, China; 5 International Associated Laboratory of Evolution and Development of Magnetotactic Multicellular Organisms (LIA-MagMC), CNRS-CAS, Qingdao 266071, China; wu@imm.cnrs.fr; 6 BGI-Qingdao, BGI-Shenzhen, Qingdao 266555, China; chenjianwei@genomics.cn; 7 Aix Marseille University, CNRS, LCB, IM2B, IMM, 13009 Marseille, France

**Keywords:** spherical MMPs, magnetite, greigite, magnetosome gene cluster, intertidal sediment

## Abstract

Multicellular magnetotactic prokaryotes (MMPs) are a unique group of magnetotactic bacteria that are composed of 10–100 individual cells and show coordinated swimming along magnetic field lines. MMPs produce nanometer-sized magnetite (Fe_3_O_4_) and/or greigite (Fe_3_S_4_) crystals—termed magnetosomes. Two types of magnetosome gene cluster (MGC) that regulate biomineralization of magnetite and greigite have been found. Here, we describe a dominant spherical MMP (sMMP) species collected from the intertidal sediments of Jinsha Bay, in the South China Sea. The sMMPs were 4.78 ± 0.67 μm in diameter, comprised 14–40 cells helical symmetrically, and contained bullet-shaped magnetite and irregularly shaped greigite magnetosomes. Two sets of MGCs, one putatively related to magnetite biomineralization and the other to greigite biomineralization, were identified in the genome of the sMMP, and two sets of paralogous proteins (Mam and Mad) that may function separately and independently in magnetosome biomineralization were found. Phylogenetic analysis indicated that the sMMPs were affiliated with *Deltaproteobacteria*. This is the first direct report of two types of magnetosomes and two sets of MGCs being detected in the same sMMP. The study provides new insights into the mechanism of biomineralization of magnetosomes in MMPs, and the evolutionary origin of MGCs.

## 1. Introduction

Magnetotactic bacteria (MTB) are a group of heterogeneous microbes that have the ability to swim along geomagnetic field lines [1,2]. The specific organelles that enable this swimming ability, termed magnetosomes, are composed of membrane-enveloped magnetite (Fe_3_O_4_) or greigite (Fe_3_S_4_) crystals. Magnetosomes arrange in chains and serve as a compass orientating the magnetotactic behavior [3]. MTB are Gram-negative bacteria displaying morphological, phylogenetical, and physiological diversity. According to the NCBI taxonomy, MTB have been found to occur in the *Alphaproteobacteria*, *Deltaproteobacteria*, *Gammaproteobacteria*, *Zetaproteobacteria*, and the candidate *Lambdaproteobacteria* and *Etaproteobacteria* classes of the *Proteobacteria* phylum (now nominated as *Pseudomonadota* phylum), the *Nitrospirae* phylum, the candidate *Omnitrophica* phylum (formerly nominated as candidate division OP3), the candidate *Latescibacteria* phylum (nominated as former candidate division WS3), and the *Planctomycetes* phylum [4,5,6,7,8,9,10,11]. Subsequently, the MTB affiliated with the *Deltaproteobacteria* could be reclassified into the *Desulfobacterota* phylum as well [12]. Recently, studies of reconstructed metagenome-assembled MTB genomes have expanded the taxonomic diversity of MTB, which could be classified into 10 phyla as defined in the NCBI taxonomy, including the *Proteobacteria*, *Nitrospirae*, *Nitrospinae*, *Planctomycetes*, *Elusimicrobia*, *Fibrobacteres*, candidate *Omnitrophica*, candidate *Latescibacteria*, candidate *Hydrogenedentes*, and candidate *Riflebacteria* [7,13,14]. Numerous morphotypes have been observed including unicellular coccoid to ovoid cells, rods, vibrios, spirilla, and a unique aggregated form termed multicellular magnetotactic prokaryotes (MMPs) or magnetoglobules [2,15].

Farina et al. (1983) initially investigated MMPs in a lagoon in Brazil. Subsequently, two distinct morphotypes of MMP have been discovered including spherical mulberry-like MMPs (sMMPs) [16,17,18,19,20,21,22,23] and ellipsoidal pineapple-like MMPs (eMMPs) [22,24,25,26,27,28]. The sMMPs comprise 10–40 cells (each 3–12 μm in diameter) that are arranged with helical symmetry. The eMMPs are composed of 28–101 cells that are arranged in ellipsoidal aggregations of 8–23 μm in length and 7–17 μm in width. Both morphotypes are able to mineralize magnetite and/or greigite magnetosomes [16,18,20,21,26,29]. The reported species and distributions of sMMPs are more diverse than for eMMPs. The sMMPs are cosmopolitan in diverse saline aquatic habitats including coastal lagoons [16,30], salt marshes [17], coastal tidal sand flats [18,31], lakes [32], intertidal zones [19,20,21,33], coral reefs [22], and mangroves [23]. The eMMPs have been reported from intertidal sediments of the Mediterranean Sea [24,27,28] and the Yellow Sea [25,26,33,34], and coral reef habitats at Drummond Island [27] and Paracel Island [22] in the South China Sea. All MMPs have been affiliated with the *Deltaproteobacteria* exclusively and show the potential for sulfate reduction [18,35].

In addition, it has been reported that the formation of magnetosomes is controlled by a magnetosome gene cluster (MGC) [9,36,37,38]. Two sets of MGCs thought to be involved in magnetite and/or greigite formation have been identified in several *Deltaproteobacteria* MTB, including two uncultured sMMPs (the greigite-producing *Candidatus* (*Ca*.) Magnetoglobus multicellularis and the magnetite- and greigite-producing *Ca*. Magnetomorum HK-1) [31,35,39], one uncultured eMMP (the magnetite-producing *Ca*. Magnetananas rongchenensis RPA) [40], and two cultured unicellular MTB (the magnetite-producing *Desulfovibrio magneticus* RS-1 and the magnetite- and greigite-producing *Desulfamplus magnetomortis* BW-1) [41,42]. The corresponding magnetosome composition and morphology were also detected in the spherical *Ca*. Magnetoglobus multicellularis (irregularly shaped greigite magnetosomes were biomineralized), unicellular *Desulfovibrio magneticus* RS-1 (bullet-shaped magnetite magnetosomes were biomineralized), and *Desulfamplus magnetomortis* BW-1 (irregularly shaped greigite and bullet-shaped magnetite magnetosomes were both biomineralized), respectively [16,42,43], except for the ellipsoidal *Ca*. Magnetananas rongchenensis RPA and the spherical *Ca*. Magnetomorum HK-1. While the RPA could biomineralize both bullet-shaped magnetite and rectangular greigite crystals, the magnetosomes in the HK-1 were not described, although it showed 99.3% identity to the spherical *Ca*. Magnetomorum rongchengroseum, which was collected in another region and is reported to produce both magnetite and greigite particles [21]. In brief, there is no direct evidence whether two sets of MGCs regulate the synthesis of the two types of magnetosomes (magnetite and greigite) in the same MMP. In this study, undertaken in the intertidal zone of Jinsha Bay (South China Sea), we found a novel sMMP that contained two paralogous magnetosome gene clusters and could concurrently biomineralize both bullet-shaped magnetite and irregularly shaped greigite magnetosomes. We sequenced the genome of this sMMP, and showed that it was more integrated than previously reported MMPs. It simultaneously contained sets of both *mam* (‘magnetosome membrane’) and *mad* (‘magnetosome associated *Deltaproteobacteria*’) gene clusters, which implied the involvement of independent processes for synthesizing the two distinct types of magnetosome.

## 2. Materials and Methods

### 2.1. Sampling and Enrichment of MMPs

Sediment samples were collected from sites in the low-tide region of the intertidal zone in Jinsha Bay (Zhanjiang City, China; 21°16.267′ N, 110°24.067′ E) on 30 August 2020 and 7 September 2021. The salinity at these sites was measured (WTW Cond 3210 SET 1; Xylem, Germany), and ranged from 19.5 to 24.2‰ during the sampling periods. Samples of the subsurface sediment and in situ seawater (approximately 1:1) were transferred to the laboratory in sterile plastic bottles, and incubated in dim light at an ambient temperature for subsequent analyses. To enrich MMPs from the sediment, each plastic bottle was shaken to mix the sediment and water, and two magnets were attached externally, one each to opposite sides of the bottle adjacent to the seawater–sediment interface [44]. MMPs attracted to the magnets were removed, and purified magnetically for a subsequent study using the modified racetrack method [45].

### 2.2. Optical and Electron Microscopy

The morphology and motility of the purified MMPs were observed using the hanging drop method using differential interference contrast (DIC) microscopy (Olympus BX51 equipped with a DP80 camera system; Olympus, Tokyo, Japan) [46]. For scanning electron microscopy (SEM) observations, each sample was fixed in 2.5% glutaraldehyde for >3 h at 4 °C, filtered onto a polycarbonate filter (high-density pores, 1 μm diameter; Whatman), dehydrated through a gradient of ethanol concentrations, dried, and gold-coated. The gold-coated samples were observed using a KYKY-2800B SEM (KYKY Technology Development Ltd., Beijing, China) operating at 25 kV. For transmission electron microscopy (TEM) observations of the MMP and magnetosomes morphologies, 10 μL samples of MMPs, which were purified using the racetrack method and concentrated by slight centrifugation, were deposited on formvar carbon-coated copper grids, washed three times with distilled water, and examined using a Hitachi HT7700 TEM (Hitachi Ltd., Tokyo, Japan) operating at 100 kV, and a JEM-2100 TEM (JEOL Ltd., Tokyo, Japan) operating at 200 kV. The composition of magnetosomes was analyzed using high-resolution transmission electron microscopy (HRTEM; JEM-2100 TEM) equipped for energy-dispersive X-ray spectroscopy (EDXS).

### 2.3. Genomic DNA Extraction, Whole Genome Amplification, and Phylogenetic Analysis of 16S rRNA Genes

The sMMPs were sorted using a TransferMan ONM-2D micromanipulator and a CellTram Oil manual hydraulic pressure-control system (IM-9B) installed on a microscope (Olympus IX51; equipped with a 40 × LD objective, Tokyo, Japan) [6,21,26,47]. The micro-sorted MMPs stored in PBS were repeatedly freeze-thawed, after which whole genome amplification (WGA) of MMPs was performed using multiple displacement amplification (MDA) for 8 h using the REPLI-g Single Cell kit (cat. #150343; Qiagen, Hilden, Germany), according to the manufacturer’s instructions [26]. The WGA products were stored at −80 °C for genome sequencing and 16S rRNA gene analysis.

The WGA products were diluted and used for amplification of the 16S rRNA gene. Universal bacterial primers 27F (5′-AGAGTTTGATCCTGGCTCAG-3′) and 1492R (5′-GGTTACCTTGTTACGACTT-3′) were used for the polymerase chain reaction (PCR) in a Mastercycler (Eppendorf, Hamburg, Germany). The purified and retrieved PCR products were cloned into the pMD18-T vector (Takara, Shiga, Japan), and transferred into competent *Escherichia coli* (strain DH5α) (Takara, Japan). Clones were selected randomly and sequenced using the vector primers M-13 and RV-M (Ruibio BioTech Co. Ltd., Qingdao, China). The 16S rDNA sequences obtained for the sMMPs were aligned against the nr/nt database using the BLAST search program (http://www.ncbi.nlm.nih.gov/BLAST/ accessed on 18 November 2021). The 16S rDNA sequences of reference MMPs and unicellular MTB were downloaded from the GenBank database. All sequences were aligned using Clustal W software version 2.1 [48], and a phylogenetic tree was constructed using the maximum likelihood (ML) method using the IQ-TREE software version 2.0.3 under the best-fit GTR+F+R4 model [49]. Bootstrap values were calculated using 1000 replicates. The tree was visualized and adjusted using iTOL webtool version 6.4.3 (https://itol.embl.de/ accessed on 26 November 2021), and was rooted with unicellular *Deltaproteobacteria* MTB.

### 2.4. Fluorescence In Situ Hybridization (FISH)

A specific oligonucleotide probe JSMW7 (5′-GCCACCTTTCATCTAATCTATC-3′) was designed for the 16S rDNA sequence corresponding to position 185–206 of the target sMMP, and its specificity was evaluated using the online probe-match tool (http://rdp.cme.msu.edu/probematch/search.jsp, accessed on 19 November 2021) [50]. The specific probe was labeled with hydrophilic sulfoindocyanine Cy3 as the fluorescent dye at the 5′ end. The universal probe EUB338 (5′-GCTGCCTCCCGTAGGAGT-3′) was used as the positive control in hybridization, and was labelled with fluorescein phosphoramidite FAM at the 5′ end. Appropriate amounts of *E. coli* cells were added to the sample and mixed with the target sMMP cells as negative controls. The specimen was treated and prepared as described previously [51,52], and FISH was carried out according to protocols reported early [51,53,54]. The hybridization results were observed using an Olympus BX51 epifluorescence microscope equipped with a DP80 camera system (Olympus, Tokyo, Japan).

### 2.5. Sequencing, Assembly, and Genome Annotation, and Comparative Analysis of Magnetosome Genes and Proteins

Paired-end 100 bp (PE100) libraries were constructed from the produced DNA of the WGA method using the MGI Easy FS DNA Library Prep Set kit (MGI, Shenzhen, China), according to the manufacturer’s instructions. The genome was sequenced using the DNBSEQ-T1 platform (BGI-Qingdao, Qingdao, China), using the paired-end 100 bp library. After quality trimming and filtering using SOAPnuke version 2.1.6 [55], the reads were assembled using MEGAHIT version 1.2.8, using k-mer sizes from 27 to 255 by step 20 [56]. Then, the metaWRAP version 1.2.1 pipeline was used for metagenome binning, refinement, and reassembly with default parameters to select the pure genome [57]. The quality of MMP genomes was assessed using QUAST version 5.0.2 [58], and genomic completeness and contamination were estimated using CheckM version 1.1.3 [59]. The genome was annotated using Prokka version 1.14.5 [60]. Several available genomes of *Deltaproteobacteria* MTB were obtained from the GenBank database, including *Desulfovibrio magneticus* RS-1 [41], *Desulfamplus magnetomortis* BW-1 [42], *Ca.* Magnetoglobus multicellularis [35,39], *Ca.* Magnetomorum HK-1 [31], and *Ca.* Magnetananas rongchenensis RPA [40]. A comparative analysis of MGCs was performed using the MagCluster version 0.2.0 [61] and the clinker [62] with a manual inspection following. The putative magnetosome proteins were confirmed using NCBI PSI-BLAST [63], and the annotations were corrected manually. Comparative analyses of putative magnetosome proteins were performed using BLASTP (http://www.ncbi.nlm.nih.gov/BLAST/, accessed on 13 December 2021).

The phylogenetic trees based on the Mam and Mad protein sequences were both constructed using the maximum likelihood (ML) method, using IQ-TREE software version 2.0.3 under the same best-fit LG+F+I+G4 model [49]. Bootstrap values were calculated using 1000 replicates. The tree was visualized and adjusted using iTOL webtool version 6.4.3 (https://itol.embl.de/, accessed on 15 January 2022).

## 3. Results

### 3.1. Occurrence, Structure, and Motility of the sMMPs

**Figure 1:**
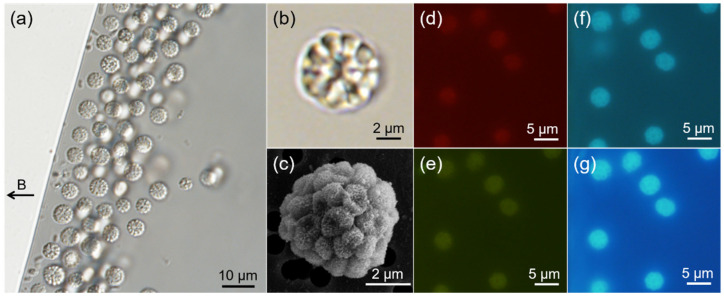
Abundance and morphology of spherical MMPs (sMMPs) from Jinsha Bay. (**a**) The sMMPs aligned to the magnetic field lines. The black arrow to the left indicates the direction of the magnetic field. Differential interference contrast image (**b**) and scanning electron micrograph (**c**) of representative sMMPs. Fluorescence images of living sMMPs illuminated by green light (**d**), blue light (**e**), violet light (**f**), and UV light (**g**).

Unicellular MTB and highly abundant sMMPs were observed in the intertidal sediment from one site in Jinsha Bay (Figure 1a). Yellow and gray layers of sand were present in the sediment, with the yellow layer approximately 1 cm above the gray layer. The sMMPs were present at a maximum abundance of approximately 304 inds./cm^3^ in the gray layer.

An analysis of DIC images (Figure 1b) and SEM micrographs (Figure 1c) showed that each sMMP contained approximately 14–40 constituent cells arranged with helical symmetry, which also appeared as a radical symmetry on the section image (*n* = 24). The sMMPs were autofluorescent when illuminated with green, blue, violet, or UV light. The cellular interfaces were evident using autofluorescence excitation at 400–410 nm and 330–385 nm wavelengths (Figure 1f,g), but the cellular contours of MMPs were not distinct when the cells were exposed to illumination at 510–550 nm and 450–480 nm (Figure 1d,e).

The average diameter of the sMMPs was 4.78 ± 0.67 μm (*n* = 237), and the average size of each individual unit was 1.03 ± 0.15 μm (*n* = 64) in the largest dimension.

**Figure 2:**
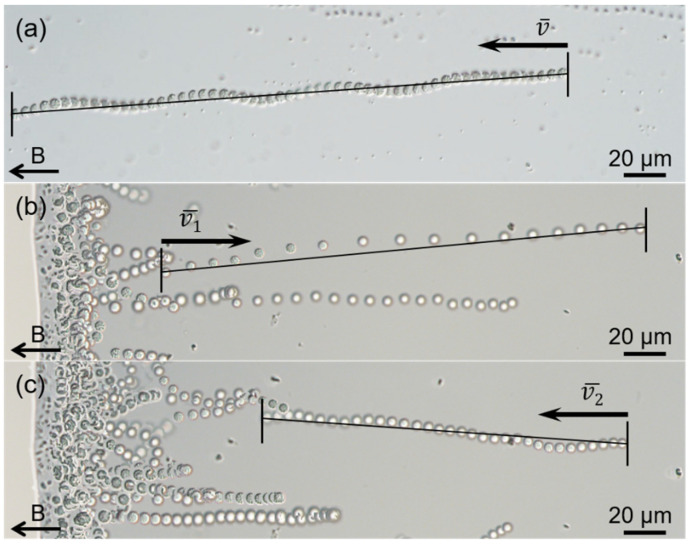
Motility of the sMMPs. Optical micrographs showing magnetotaxis motion (**a**), and excursion (**b**) and return (**c**) of the “ping-pong” motility. The black arrows at the bottom left of each image indicate the direction of the magnetic field. The swimming of the sMMPs was recorded at 20 frames per second (FPS).

Use of the hanging drop method showed that >80% of magnetically enriched sMMPs exhibited north-seeking polarity (Figure 1a), and swam along the magnetic field lines with an average speed of approximately 78.0 ± 41.4 μm/s ($\bar{v}$, *n* = 38; maximum velocity, 177.4 μm/s) along a straight or helical trajectory (Figure 2a). Typical “ping-pong” motility was also observed at droplet edges, the north-seeking sMMPs which accumulated at the edge showed an excursion swim against the magnetic field lines away from the droplet edge (Figure 2b), then performed a return swim along the magnetic field lines back to the droplet edge (Figure 2c). Additionally, the average speeds of excursion and return were 223.9 ± 54.5 μm/s (${\bar{v}}_{1}$, *n* = 24; maximum velocity, 330.5 μm/s) and 102.2 ± 19.0 μm/s (${\bar{v}}_{2}$, *n* = 24; maximum velocity, 138.4 μm/s), respectively (Figure 2b,c).

### 3.2. Characterization of Magnetosome Biomineralization in the sMMPs

**Figure 3:**
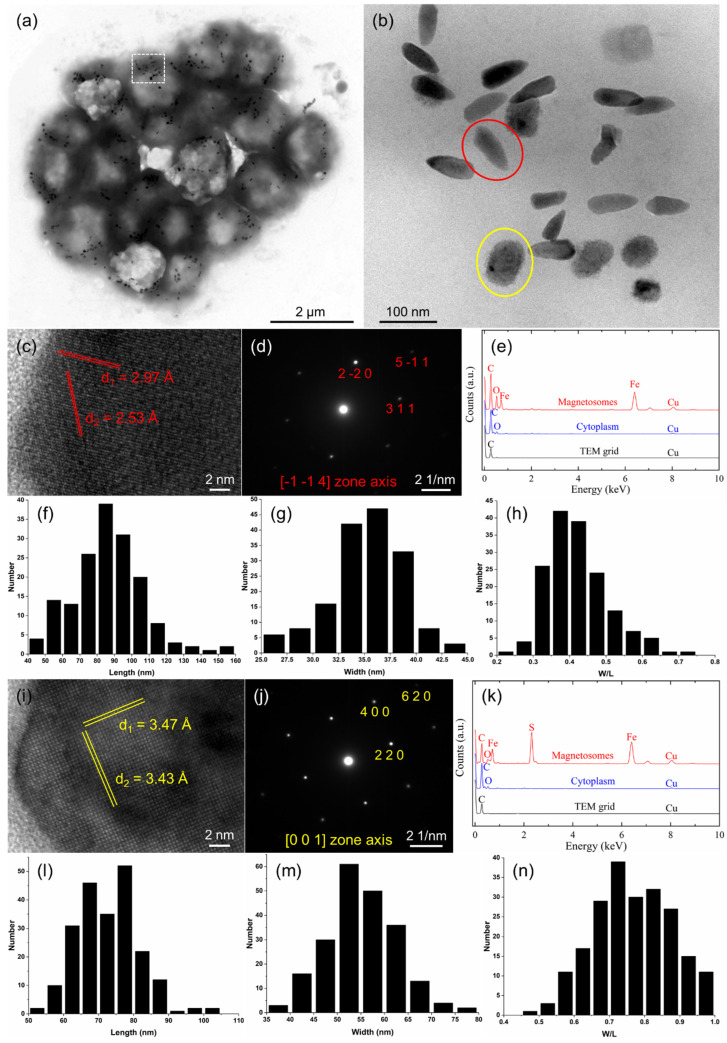
Morphology and characteristics of the magnetosomes of an sMMP cell from Jinsha Bay. (**a**) Transmission electron micrograph of an sMMP cell. (**b**) The enlarged image of the white rectangle in the image (**a**), showing bullet-shaped and irregularly shaped magnetosomes in red and yellow circles, respectively. (**c**) High-resolution transmission electron microscopy (HRTEM) image of a bullet-shaped magnetosome. (**d**) The corresponding indexed Fast Fourier transform pattern of the bullet-shaped particle shown in (**c**). (**e**) Energy-dispersive X-ray spectroscopy (EDXS) analysis of a bullet-shaped crystal. Histograms of the length (**f**), width (**g**), and width/length ratio (**h**) of bullet-shaped magnetite magnetosomes. (**i**) HRTEM image of an irregularly shaped magnetosome. (**j**) The corresponding indexed Fast Fourier transform pattern of the irregularly shaped particle shown in (**i**). (**k**) EDXS analysis of an irregularly shaped crystal. Histograms of the length (**l**), width (**m**), and width/length ratio (**n**) of the irregularly shaped greigite magnetosomes.

TEM observations showed the simultaneous presence of both bullet-shaped and irregularly shaped magnetosomes arranged in chains or clusters within constituent cells of the sMMPs from the South China Sea (Figure 3a,b). The average number of magnetosomes per individual cell was 30 ± 11 (*n* = 31), with the proportions of bullet-shaped (Figure 3b, red circle) and irregularly shaped crystals (Figure 3b, yellow circle) averaging 43.3% and 56.7%, respectively. HRTEM and EDXS analyses indicated that the bullet-shaped magnetosomes were composed of magnetite (Fe_3_O_4_) (Figure 3c–e). The magnetite particles were 87.0 ± 20.3 × 35.2 ± 3.5 nm in size and had a width/length ratio of 0.42 ± 0.08 (*n* = 163) (Figure 3f–h). These analyses (HRTEM and EDXS) also showed that the irregularly shaped crystals were composed of greigite (Fe_3_S_4_) (Figure 3i–k). The greigite particles were 72.8 ± 8.7 × 55.2 ± 7.3 nm in size and had a width/length ratio of 0.77 ± 0.11 (*n* = 215) (Figure 3l–n).

### 3.3. Phylogenetic Analysis of the sMMPs

We isolated two sMMPs using the micromanipulation sorting method, and their genomic DNA was extracted and amplified by WGA using MDA. The 16S rRNA genes were amplified, cloned, and sequenced from the WGA product. Sequences (53) related to the MMPs were obtained from 55 randomly chosen clones. All the MMP sequences shared an identity of at least 99.1%, indicating that the sMMPs represented a single species. FISH was used to corroborate the authenticity of the 16S rRNA gene sequences. Fluorescence microscopy observations showed that all bacterial cells were hybridized with the general probe EUB338 (Figure S1a, green), while only the spherical MMP cells were hybridized with the specific probe JSMW7, designed from the 16S rRNA sequence of the sMMP (Figure S1b, red). These results demonstrated the specificity of the FISH analysis.

**Figure 4:**
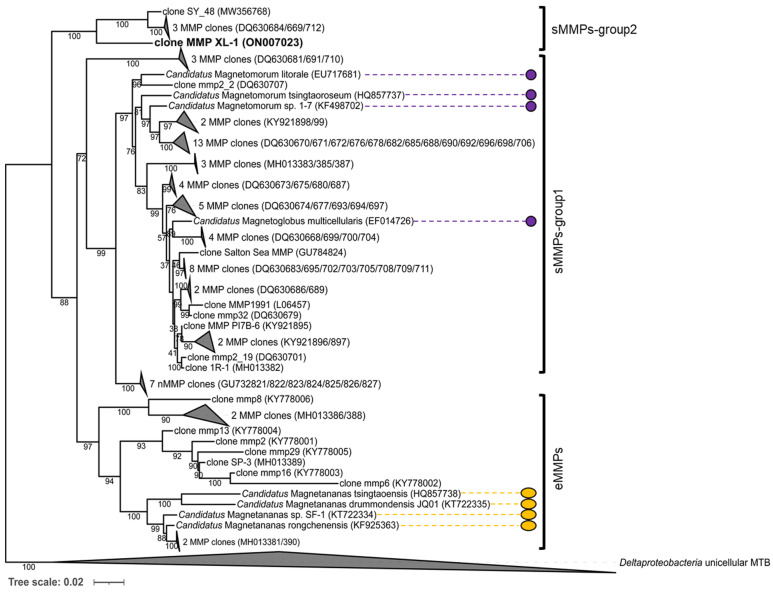
Phylogenetic tree based on 16S rRNA gene sequence analysis. The phylogenetic tree was constructed using the maximum likelihood method using IQ-TREE software. Bootstrap values were calculated using 1000 replicates. The acquired sMMP sequence is indicated in bold. GenBank accession numbers are shown in parentheses. The scale bar represents 2% sequence divergence. The morphologies and characteristics of the sMMPs (labeled by the purple circles) and the eMMPs (labeled by the orange ellipses) have been described.

The 16S rRNA gene sequence of the sMMP was most closely related (93.3% shared sequence identity) to that of the uncultured delta proteobacterium clone SY_48 (MW356768), which was collected from a mangrove area in Sanya [23]. It also showed 6.8–7.0% sequence divergence from the uncultured delta proteobacteria clones mmp2_9 (DQ630712), mmp45 (DQ630684), and mmp12 (DQ630669), from the Little Sippewissett salt marshes in Falmouth [17]. The phylogenetic analysis of the 16S rRNA gene sequence revealed that these five sMMP clones formed another group of spherical-type MMPs, and belong to the *Deltaproteobacteria* (Figure 4). We tentatively designate this novel isolated sMMP from Jinsha Bay at Zhanjiang City as MMP XL-1 (Figure 4).

### 3.4. General Genomic Features of the Proposed MMP XL-1 and Comparative Genomic Analysis of Magnetosome Gene Clusters

Approximately 8.88 μg of genomic DNA was obtained from the two micro-sorted sMMPs following WGA. Following sequencing, assembly, and binning, a draft genome of approximately 8.49 Mb in size and having a GC content of 34.6% was obtained. The genome contained 279 contigs and had a 60,219 bp N50-value. A total of 5329 coding sequences (CDS) were predicted and annotated, including 46 tRNAs, one tmRNA, and one complete rRNA gene operon. The estimated completeness and contamination of the genome were 97.6% and 1.6%, respectively.

**Figure 5:**
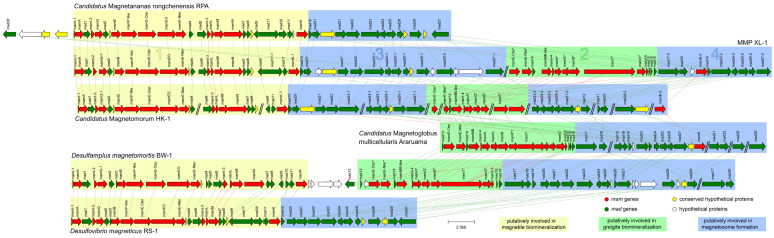
Comparison of MGCs from MMP XL-1 and other previously reported genomes, including *Ca*. Magnetananas rongchenensis RPA, *Ca*. Magnetomorum HK-1, *Ca*. Magnetoglobus multicellularis Araruama, *Desulfamplus magnetomortis* BW-1, and *Desulfovibrio magneticus* RS-1. The bars between clusters indicate homologous genes. The magnetosome genes putatively involved in magnetite biomineralization are enclosed by yellow, those putatively involved in greigite biomineralization are enclosed by green, and those putatively involved in magnetosome formation are enclosed by blue.

Two sets of MGCs were separately identified in contigs XL1_145 and XL1_87 in the genome (Table S1). On contig XL1_145, the MGC-related region had a size of 40,808 bp (Figure 5, clusters 1 and 3) and the GC content was 35.6%. The region included 14 *mam* genes, 20 *mad* genes, and others. The set of magnetosome genes on cluster 1, including *mamI-1*, *A*, *I-2*, *Q-1*, *B*, *P-like*, *E-Cter*, *EO*, *E-Nter*, *I-3*, *L*, *M*, *N*, *K-1*, and *mad1*, *2*, *4*, *6*, *7*, *8*, *9*, *17-1*, *11* (termed the *mamAB-like* operon), had a similar gene order and high similarity to the magnetite gene clusters in *Ca.* Magnetomorum HK-1, *Ca.* Magnetananas rongchenensis RPA, and *Desulfamplus magnetomortis* BW-1, which indicates that these genes are most likely related to the putative magnetite magnetosome biomineralization (Figure 5, cluster 1) [31,40,64].

On contig XL1_87, the MGC-related region had a size of 24,835 bp (Figure 5, clusters 2 and 4) and the GC content was 35.1%. It included 11 *mam* genes, 12 *mad* genes, and a hypothetical gene. Another set of magnetosome genes on cluster 2 (termed the mamAB-like^*^ operon) contained mamE-Cter^*^, E-Nter^*^, *MB-like*, *I-4*, A^*^, Q^*^, B^*^, T^*^, O^*^, *I-5*, *K-2*, and *mad14*, *15*, *15’*, *16* in order. The arrangement and identities of these magnetosome genes were most similar to the corresponding genes of greigite-producing *Deltaproteobacteria*, including *Ca.* Magnetomorum HK-1, *Ca.* Magnetoglobus. multicellularis, and *Desulfamplus magnetomortis* BW-1 [31,35,39,64], indicating that these genes are probably responsible for putative greigite magnetosome biomineralization (Figure 5, cluster 2).

A total of 32 *mad* genes were identified in the magnetosome gene map; besides some of these located in the two *mamAB-like* operons, there were still two *mad* gene clusters found downstream of the magnetite gene cluster (including *mad10*, *31*, *21*, *22*, *23-1*, *24-1*, *25-1*, *26-1*, *28-1*, *28-2*, and *27-1*) (Figure 5, cluster 3) and the greigite gene cluster (including *mad17-2*, *30*, *19*, *23-2*, *24-2*, *25-2*, *26-2*, and *27-2*) (Figure 5, cluster 4), which were termed cluster 3 and cluster 4, respectively.

**Figure 6:**
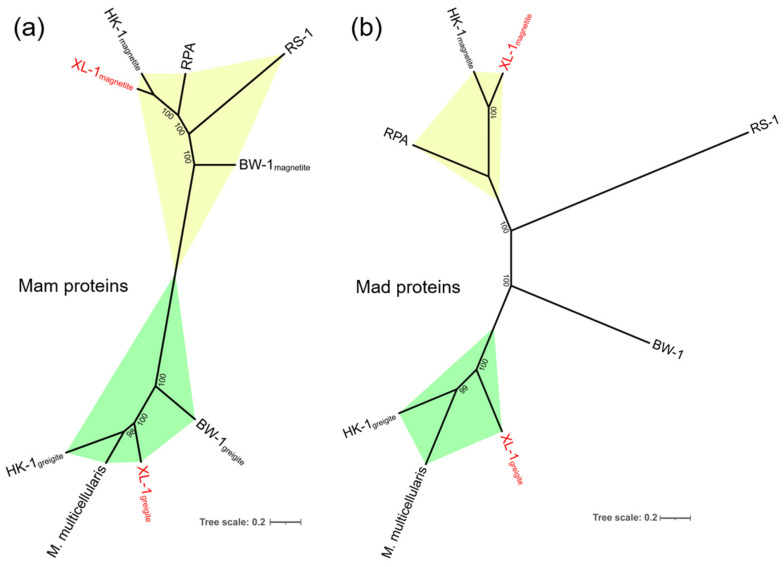
Phylogenetic tree based on (**a**) seven concatenated magnetosome membrane protein sequences (MamA*B*E-Cter*E-Nter*I*K*Q) and (**b**) five concatenated magnetosome-associated *Deltaproteobacteria* protein sequences (Mad23*24*25*26*27) that reflect the evolution of magnetotaxis. The trees were constructed using the maximum likelihood method, using IQ-TREE software. Bootstrap values were calculated using 1000 replicates. The scale bar represented 20% sequence divergence. Six strains were chosen, including *Ca.* Magnetomorum (HK-1), *Ca.* Magnetananas rongchenensis (RPA), *Desulfovibrio magneticus* (RS-1), *Desulfamplus magnetomortis* (BW-1), *Ca.* Magnetoglobus multicellularis (*M. multicellularis*), and MMP (XL-1) (acquired in this study and shown in red). The proteins involved in magnetite and greigite biomineralization are shown in the yellow and green regions, respectively.

The 11 homologous Mam proteins in clusters 1 and 2 had identities ranging from 46.3% to 83.8% (Table 1, in red), and the six homologous Mad proteins detected in clusters 3 and 4 showed identities between 52.1% and 72.2% (Table 1, in black). There may be two sets of homologous magnetosome gene clusters in the genome of sMMP from Jinsha Bay, one responsible for magnetite magnetosome biomineralization (clusters 1 and 3), and another associated with greigite magnetosome biomineralization (clusters 2 and 4) (Table 1 and Figure 5). A strong correlation in the phylogenies involved in magnetosome biomineralization was found, based on the Mam and Mad protein amino acid sequences (Figure 6). In the phylogenetic tree of Mam proteins, the magnetite-related Mam proteins (including these from XL-1, HK-1, RPA, RS-1, and BW-1) were gathered in a single branch, while greigite-related Mam proteins from HK-1, *Ca.* Magnetoglobus multicellularis, XL-1, and BW-1 were gathered in a different branch (Figure 6a). Similar clades were present in the phylogenetic tree of Mad proteins. The Mad proteins from XL-1, HK-1, and RPA involved in magnetite biomineralization were clustered in one branch, while the Mad proteins involved in greigite biomineralization were clustered in another branch, including these detected in HK-1, *Ca.* Magnetoglobus multicellularis, and XL-1 (Figure 6b).

## 4. Discussion

In this study, we isolated a dominant type of sMMP from the intertidal sediments of Jinsha Bay, in the South China Sea. Phylogenetically, the sMMPs could be classified into a novel species of *Deltaproteobacteria* using 16S rRNA analysis strongly, which had less than 98.65% 16S rRNA gene sequence identity with its closest species [65]. Each aggregate contained approximately 14–40 constituent cells and the individual cells of these sMMPs were arranged loosely in helical symmetry, which showed a little difference from the tight arrangement of other previously reported sMMPs [16,18,20,21]. Their diameter was approximately 4.78 μm, smaller than most reported sMMPs, except for the sMMPs (4.6 μm average diameter) collected from the mangrove habitat in the Sanya River (Table 2) [16,18,20,21,23,66]. The velocity of the magnetotaxis motility and the ping-pong motion of the MMP XL-1 was more similar to that of the ellipsoidal *Ca.* Magnetananas rongchenensis than to other sMMPs (Table 2) [26]. Both bullet-shaped magnetite and irregularly shaped greigite crystals were simultaneously biomineralized by this sMMP (Figure 3), which is a phenomenon previously observed in sMMPs from Itaipu Lagoon (Brazil), Lake Yuehu (China), and the Sanya mangroves (China) (Table 2) [21,23,67]. It has been reported that MTB show a clear vertical distribution [33] in the oxic–anoxic interface zone and/or anoxic regions [2]. Magnetite-producing MTB usually inhabit the top of the oxic–anoxic interface, whereas greigite-producing MTB prefer the reducing environment at the base and slightly below the interface [5,68]; this implies that those sMMPs that can biomineralize both magnetite and greigite may have broader vertical niches.

The characteristics described above show that MMP XL-1 represented a unique type of sMMP; consequently, it was further investigated using genomic studies. To date, only two draft genomes of sMMPs were obtained, including the *Ca.* Magnetoglobus multicellularis and *Ca.* Magnetomorum HK-1 [31,35]. The genome of *Ca.* Magnetoglobus multicellularis was the first MMP genome to be analyzed; this showed that the genes involved in magnetite biomineralization were homologous with the genes in greigite-producing MTB, suggesting that the magnetotactic trait is monophyletic [35,39]. Two sets of magnetosome genes, one each responsible for magnetite and greigite biosynthesis, were identified in HK-1, providing the first evidence that two divergent magnetosome gene clusters can co-occur in a single MMP genome [31].

Subsequently, comparative genomic analysis was carried out to clarify which genes within XL-1 governed the biomineralization of each magnetite and greigite. Two sets of relatively complete magnetosome gene clusters, located on two contigs, were recognized in the genome (Table S1). One set showed synteny with homologous regions for the magnetite MGC of *Ca.* Magnetomorum HK-1 and the magnetite-producing *Ca.* Magnetananas rongchenensis RPA, while another set had a higher similarity with the MGC cluster from the greigite-producing *Ca.* Magnetoglobus multicellularis and the greigite MGC of *Ca.* Magnetomorum HK-1 (Figure 5) [31,39,40]. In addition, two sets of *mam* genes involved in magnetite and greigite formation were detected in the unicellular *Desulfamplus magnetomortis* BW-1, and these were homologous with those of MMP XL-1 [42,64]. Synteny of the MGCs between this sMMP (MMP XL-1) and other *Deltaproteobacteria* bacteria is conserved, providing strong evidence that magnetite and greigite magnetosomes can be biomineralized by XL-1, controlled by two sets of MGCs (Figure 5). This is the first direct report that an MMP can contain two types of magnetosomes and corresponding MGCs (Table 2). Only greigite crystals and greigite-related genes were identified in *Ca.* Magnetoglobus multicellularis [16,35,39]. Although two types of MGCs were detected in the spherical *Ca.* Magnetomorum HK-1, the description of the magnetosome particles was not included [31]. Among eMMPs, only for one species (*Ca.* Magnetananas rongchenensis RPA) has MGC information been reported to date, and its involvement was limited to magnetite formation [26,40].

We also found that the proteins encoding magnetite and greigite biomineralization were orthologous in MMP XL-1 and other *Deltaproteobacteria* bacteria (Table S2). The magnetite proteins of XL-1 shared 52.3–96.6%, 63.5–91.5%, 56.3–86.0%, and 46.3–84.3% amino acid similarities to the corresponding proteins from HK-1, RPA, BW-1, and RS-1, respectively. The greigite proteins of XL-1 showed 60.2–90.1%, 65.9–88.6%, and 49.7–85.8% amino acid similarities to the corresponding proteins from HK-1, *Ca.* Magnetoglobus multicellularis, and BW-1, respectively (Table S2) [31,35,39,40,41,64]. This provides further evidence that supports the presence of two types of magnetosomes within XL-1.

Intriguingly, we found that two sets of genes coding for proteins related to magnetite and greigite formation within XL-1 were paralogous. They shared variable degrees of amino acid similarity 46.3–83.8% (Table 1). The similarity between its own magnetite and greigite proteins was smaller than that between its magnetite proteins and corresponding magnetite proteins of other *Deltaproteobacteria* MTB. Additionally, a similar phenomenon was shown when using the greigite proteins for comparison (Table S2), which was consistent with the similarity difference of MGCs of the *Ca.* Magnetomorum HK-1. The greigite genes of HK-1 were more congruent with the greigite genes of other MTB than with its own magnetite genes [31]. This finding may be consistent with the hypothesis that the occurrence of two sets of magnetosome genes in *Deltaproteobacteria* MTB may have originated from ancient gene duplication and/or mutation in a common ancestor, then distributed to various MTB by HGT [47], and subsequent vertical inheritance by descent [31,70,71].

It is noteworthy that the arrangement and identities of magnetosome genes on clusters 1 and 3 of XL-1 were similar to the corresponding magnetite genes of RPA. The set of magnetosome genes on the clusters 2 and 4 of XL-1 had a similar gene order and high similarity to the greigite gene clusters of *Ca.* Magnetoglobus multicellularis, which implied that the clusters 1 and 3 were involved in the magnetite biomineralization, while clusters 2 and 4 were responsible for the greigite biomineralization within XL-1, respectively (Figure 5). Additionally, the degree of the paralogous Mam and Mad proteins similarities mentioned above was almost the same (46.3–83.8% of the Mam proteins and 52.1–72.2% of the Mad proteins) (Table 1). Furthermore, the clades in the phylogenetic trees based on the Mam and Mad protein sequences were closely consistent as well (Figure 6), suggesting these two sets of *mad* genes (*mad23*, *mad24*, *mad25*, *mad26*, and *mad27*) were likely involved in the magnetite and greigite separately, which were the same as the *mam* genes (Figure 5, clusters 3 and 4) [31,42,64]. The short gene cluster that included *mad23*, *mad24*, *mad25*, and *mad26* has also been identified in all *Nitrospirae* MTB that synthesize bullet-shaped magnetosomes [72,73], which implies that they are also the core genes for magnetosome biomineralization. In addition, two branches were readily distinguishable on each of the Mam and Mad phylogenetic trees; one of these was involved in magnetite biomineralization and the other with greigite biomineralization (Figure 6). This provides further evidence for the presence of two sets of MGCs within MMP XL-1.

## 5. Conclusions

In this study, we firstly isolated sMMPs from the intertidal sediments of Jinsha Bay, in the South China Sea. Using TEM and EDXS analyses, we showed that both bullet-shaped magnetites and irregularly shaped greigite crystals were biomineralized within one spherical multicellular aggregation. SEM observations indicated that individual units within the aggregation were arranged with radial symmetry. Phylogenetic analysis showed that the sMMPs formed a new clade with four other MMP clones (MW356768, DQ630712, DQ630684, and DQ630669), and it was affiliated to the *Deltaproteobacteria*. Two sets of MGCs involved in magnetite and greigite biomineralization were identified in the genome, and these were found to be more integrated than what has previously been reported in MMPs. The two *mamAB*-like operons coupled with the downstream *mad* gene cluster have the potential to control magnetosome biomineralization (magnetite or greigite). It has displayed direct evidence that both magnetosome morphologies and the MGCs related to the two types of magnetosome co-occur in the same species, which provides new information relevant to the study of biomineralization mechanisms associated with the magnetosomes of MMPs, and the evolutionary origin of MGCs.

## Figures and Tables

**Table 1:** Comparison of Mam and Mad proteins putatively controlling the biomineralization of magnetite and greigite magnetosomes of MMP XL-1.

Proteins in Magnetite Biomineralization	Accession Number	Proteins in Greigite Biomineralization	Accession Number	Identity (%)	E-Value
MamI-1	XL1_145_00043	MamI-4	XL1_87_00005	62.50	2.68e-06
MamA	XL1_145_00042	MamA *	XL1_87_00006	46.34	8.62e-19
MamI-2	XL1_145_00040	MamI-5	XL1_87_00011	64.10	7.65e-07
MamQ-1	XL1_145_00039	MamQ *	XL1_87_00007	46.28	3.82e-18
MamB	XL1_145_00036	MamB *	XL1_87_00008	58.93	1.22e-61
MamP-like	XL1_145_00035	MamT *	XL1_87_00009	55.75	1.65e-14
MamE-Cter	XL1_145_00034	MamE-Cter *	XL1_87_00001	52.70	4.36e-23
MamEO	XL1_145_00033	MamO *	XL1_87_00010	61.54	1.31e-49
MamE-Nter	XL1_145_00032	MamE-Nter *	XL1_87_00002	65.84	1.24e-42
MamM	XL1_145_00026	MamMB-like	XL1_87_00004	48.43	2.86e-21
Mad17-1	XL1_145_00020	Mad17-2	XL1_87_00015	72.15	0
MamK-1	XL1_145_00018	MamK-2	XL1_87_00018	83.81	1.73e-179
Mad23-1	XL1_145_00011	Mad23-2	XL1_87_00020	58.28	1.23e-95
Mad24-1	XL1_145_00010	Mad24-2	XL1_87_00021	52.12	9.87e-14
Mad25-1	XL1_145_00009	Mad25-2	XL1_87_00022	62.87	1.12e-47
Mad26-1	XL1_145_00008	Mad26-2	XL1_87_00023	52.73	4.27e-10
Mad27-1	XL1_145_00001	Mad27-2	XL1_87_00024	71.36	7.13e-156

Mam proteins are shown in red, Mad proteins are shown in black. * Putatively involved in greigite biomineralization.

**Table 2:** Comparative characteristics of *Deltaproteobacteria* MTB.

Morphology of MTB	Sampling Site	Cell Diameter/Size (μm)	Magnetotaxis Motility (μm/s)	“Ping-Pong” Motility		Magnetosome Characteristics					MGC	Detected sMMP Strain	References
				Excursion (μm/s)	Return (μm/s)	Type	Shape	Composition	Size (nm)	Proportion of Magnetite (%)			
sMMP	Itaipu Lagoon, Brazil (43°04′ W, 22°57′ S)	-	-	-	-	Type-I	Bullet-shaped	Magnetite	104 ± 29 (L) 42 ± 6 (W)	-	-	-	[67]
						Type-II	Bullet-shaped	Magnetite	95 ± 23 (L) 38 ± 5 (W)	15–96			
							Irregularly shaped	Greigite	72 ± 8 (L)				
						Type-III	Irregularly shaped	Greigite	70 ± 8 (L)	-			
	Araruama Lagoon, Brazil (42°13′ W, 22°50′ S)	6.0–9.5	90 ± 20	-	-	Type-III	Irregularly shaped	Greigite	88 (L) × 71 (W)	-	Greigite biomineralization	*Ca*. Magnetoglobus multicellularis (EF014726)	[16,39]
	Wadden Sea, northern Germany (53°53.555′ N, 8°40.565′ E)	5.7 ± 1.1	-	-	-	Type-IV	Bullet-shaped	Greigite	91 ± 21 (L) 40 ± 6 (W)	-	-	*Ca*. Magnetomorum litorale (EU717681)	[18]
	Wadden Sea, northern Germany (53°53.53′ N, 8°40.75′ E)	-	-	-	-	-	-	-	-	-	Magnetite and greigite biomineralization	*Ca*. Magnetomorum HK-1 (GCA_001292585)	[31]
	Yuehu Lake, China (37°21′ N, 122°33′ E)	5.5 ± 0.8	-	-	-	Type-I	Bullet-shaped	Magnetite	92 ± 27 (L) 29 ± 5 (W)	-	-	-	[19]
		5.6 ± 0.9	37 ± 20	124 ± 53	93 ± 39	Type-II	Bullet-shaped	Magnetite	80.1 ± 16.1 (L) 33.6 ± 3.5 (W)	21.8–64.8	-	*Ca*. Magnetomorum rongchengroseum (KF498702)	[21]
							Irregularly shaped	Greigite	63.9 ± 9.3 (L) 52.5 ± 7.5 (W)				
	Huiquan Bay, China (36°03′ N, 120°21′ E)	5.5 ± 0.8	55	-	-	Type-I	Bullet-shaped	Magnetite	92 ± 20 (L) 35 ± 4 (W)	-	-	*Ca*. Magnetomorum tsingtaoroseum (HQ857737)	[20]
	Sanya Mangrove, China (18°15.242′ N, 109°30.585′ E)	4.6 ± 0.2	-	-	-	Type-I	Bullet-shaped	Magnetite	78 ± 18 (L) 34 ± 4 (W)	-	-	SY_5 (MW356767) SY_48 (MW356768)	[23]
						Type-II	Bullet-shaped	Magnetite	88 ± 19 (L) 34 ± 5 (W)	-			
							Irregularly shaped	Greigite	80 ± 19 (L)				
						Type-III	Irregularly shaped	Greigite	77 ± 11 (L)	-			
	Jinsha Bay, China (21°16.267′ N, 110°24.067′ E)	4.78 ± 0.67	78.0 ± 41.4	223.9 ± 54.5	102.2 ± 19.0	Type-II	Bullet-shaped	Magnetite	87.0 ± 20.3 (L) 35.2 ± 3.5 (W)	8.2–82.0	Magnetite and greigite biomineralization	MMP XL-1	This study
							Irregularly shaped	Greigite	72.8 ± 8.7 (L) 55.2 ± 7.3 (W)				
eMMP	Huiquan Bay, China (36°03′ N, 120°21′ E)	9.6 ± 1.2 × 7.8 ± 0.9	99 ± 50	-	-	Type-I	Bullet-shaped	Magnetite	102 ± 24 (L) 38 ± 6 (W)	-	-	*Ca*. Magnetananas tsingtaoensis (HQ857738)	[25]
	Yuehu Lake, China (37°21′ N, 122°34′ E)	9.18 ± 1.01 × 7.41 ± 0.76	77 ± 33	223 ± 27	169 ± 27	Type-I	Bullet-shaped	Magnetite	115 ± 27 (L) 39 ± 5 (W)	-	Magnetite biomineralization	*Ca*. Magnetananas rongchenensis (KF925363)	[26,40]
						Type-II	Bullet-shaped	Magnetite	-				
							Irregularly shaped	Greigite	102 ± 14 (L) 78 ± 13 (W)				
Rod-shaped	Badwater Basin, America	-	30	-	-	Type-II	Bullet-shaped	Magnetite	-	-	Magnetite and greigite biomineralization	*Desulfamplus magnetovallimortis* BW-1 (JN252194)	[42,69]
							Irregularly shaped	Greigite	-				
Vibrio-shaped	Kameno River waterway, Japan	3–5 × 1	-	-	-	Type-I	Bullet-shaped	Magnetite	-	-	Magnetite biomineralization	*Desulfovibrio magneticus* RS-1 (NR_027575)	[41,43]

“L” and “W” indicate the length and width of magnetosome crystals, respectively. “-” means no data acquired.

## Data Availability

Publicly available datasets were analyzed in this study. This data can be found here: The 16S rRNA gene and magnetosome gene sequences of MMP XL-1 have been deposited in GenBank under the accession numbers ON007023, ON204283, and ON204284, respectively. Additionally, reported genome sequences of *Desulfovibrio magneticus* RS-1, *Desulfamplus magnetomortis* BW-1, *Ca.* Magnetoglobus multicellularis, *Ca.* Magnetomorum HK-1, and *Ca.* Magnetananas rongchenensis RPA have been acquired from GenBank under the accession numbers NC_012796, GCF_900170035, GCF_000516475, GCA_001292585, and KY084568, respectively.

## Supplementary Materials

The following supporting information can be downloaded at: https://www.mdpi.com/article/10.3390/microorganisms10050925/s1, Figure S1: Identification of the sMMPs based on fluorescence in situ hybridization (FISH) analyses; Table S1: General features of the contigs, including the magnetosome gene clusters of MMP XL-1; Table S2: Comparison of Mam and Mad proteins involved in magnetite and greigite biomineralization in MMP XL-1 with those of *Ca.* Magnetomorum HK-1, *Ca.* Magnetananas rongchenensis RPA, *Ca.* Magnetoglobus multicellularis, *Desulfamplus magnetomortis* BW-1, and *Desulfovibrio magneticus* RS-1.
