# Supplementary figures and images for: A Novel Isolate of Spherical Multicellular Magnetotactic Prokaryotes Has Two Magnetosome Gene Clusters and Synthesizes Both Magnetite and Greigite Crystals
Source: Microorganisms. 2022 Apr 28;10(5):925. doi: 10.3390/microorganisms10050925 (PMC9145555; doi:10.3390/microorganisms10050925)

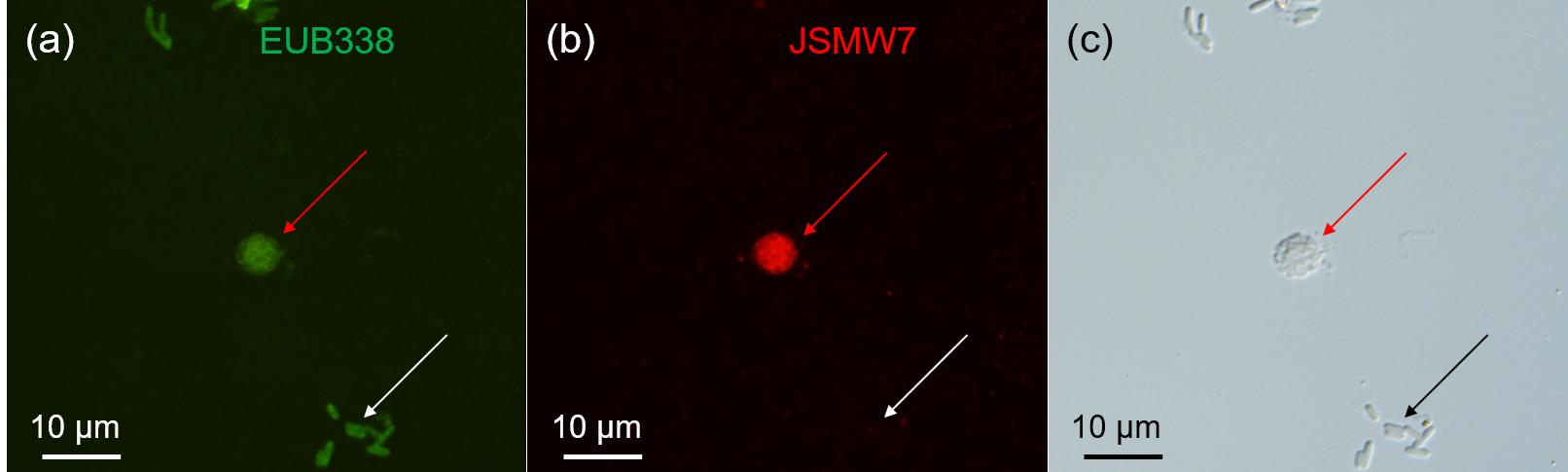

Supplement: Supplementary file 1 [file microorganisms-10-00925-s001.zip › Figure S1. Identification of the sMMPs based on fluorescence in situ hybridization (FISH) analyses.tif]
